# *Doenjang*, A Korean Traditional Fermented Soybean Paste, Ameliorates Neuroinflammation and Neurodegeneration in Mice Fed a High-Fat Diet

**DOI:** 10.3390/nu11081702

**Published:** 2019-07-24

**Authors:** Je Won Ko, Young-Shin Chung, Chung Shil Kwak, Young Hye Kwon

**Affiliations:** 1 Department of Food and Nutrition, Seoul National University, Seoul 08826, Korea; 2 Department of Biotechnology and The Research Institute for Basic Sciences, Hoseo University, Asan, Chungnam 31499, Korea; 3 Institute on Aging, Seoul National University, Seoul 03080, Korea; 4 Research Institute of Human Ecology, Seoul National University, Seoul 08826, Korea

**Keywords:** fermentation, high-fat diet, isoflavone, neurodegenerative disease, neuroinflammation, soybean

## Abstract

Obesity is considered a risk factor for neurodegeneration. Because fermentation of soybean increases contents of various bioactive compounds with anti-obesity and anti-diabetic activities, we investigated the protective effect of *doenjang*, a Korean traditional fermented soybean paste, against neuroinflammation and neurodegeneration in the cortex and hippocampus of mice fed a high-fat (HF) diet. C57BL/6J mice were fed a low-fat diet, an HF diet, an HF-containing steamed soybean diet, or an HF-containing *doenjang* (DJ) diet for 11 weeks. *Doenjang* consumption alleviated hippocampal neuronal loss, which was increased by the HF diet. Accordingly, we observed higher cell proliferation and neurotrophic factor mRNA levels in the DJ group. Contents of oxidative metabolites and mRNA levels of oxidative stress- and neuroinflammation-related genes were lower in the DJ group compared to the HF group. Dietary *doenjang* reduced β-amyloid peptide (Aβ) levels by regulating gene expressions involved in Aβ production and degradation. Furthermore, *doenjang* consumption reduced tau hyperphosphorylation induced by HF feeding. Overall, *doenjang* was more effective than steamed soybean in suppressing neuroinflammation and neurodegeneration in mice fed an HF diet. These results suggest that bioactive compounds produced during the fermentation and aging of soybean may be involved in the enhanced neuroprotective effects of *doenjang*.

## 1. Introduction

Obesity is considered as a major risk factor for the development of type 2 diabetes, non-alcoholic fatty liver disease (NAFLD), and cardiovascular disease [1]. Furthermore, accumulating evidence suggest that obesity is causally linked to neurodegenerative diseases [2]. Due to the possible cross-talk between peripheral tissues and the brain, chronic inflammation and insulin resistance may play important roles in inducing brain dysfunction such as altered synaptic plasticity and cognitive impairment [3,4,5]. Mechanistically, obesity-related neuronal stress and neuroinflammation was attributed to disruption of the blood-brain barrier, infiltration of immune cells, and activation of microglial cells [3]. Because of high metabolic rates of oxygen consumption and high levels of polyunsaturated fatty acids, neurons are known to be more susceptible to oxidative stress than other organs [6].

Soybean and soy products are a particularly rich source of isoflavone with antioxidant and phytoestrogenic activities, which contribute to their beneficial effects on lipid metabolism, bone development, and cardiovascular and central nervous systems [7]. In soybean and soy foods, isoflavones are contained in either aglycones, such as genistein, daidzein, and glycitein, or their respective β-glycosides, such as genistin, daidzin, and glycitin. The absorption of aglycones is faster and more extensive than that of the glycosides; therefore, isoflavone aglycone-rich products may provide additional health benefits over glucoside-rich products [8]. Physiologically relevant levels of isoflavone are shown to mimic beneficial effects of 17β-estradiol on the regulation of neuronal viability, β-amyloid peptide (Aβ) accumulation, and tau hyperphosphorylation [9].

Our previous studies reported that genistein significantly inhibited inflammation in the liver of *db/db* mice fed a methionine-choline-deficient diet [10] and prevented NAFLD and neurodegeneration of ApoE knock-out mice fed a high-fat (HF) diet [11,12]. In addition, we have reported the neuroprotective effect of genistein against endoplasmic reticulum (ER) stress-mediated neurotoxicity in SH-SY5Y neuroblastoma cells [13]. Others have reported that isoflavone has recovered the cognition deficit induced in Aβ-injected rats [14,15], in the mice model of Parkinson’s disease [16], and in streptozotocin-induced diabetic mice [17].

In *doenjang*, a traditional Korean fermented soybean paste, the qualitative and quantitative composition of soybean components are significantly changed by enzymatic processes during fermentation. Meanwhile, glycosylated isoflavone converts into aglycones with greater anti-obesity and anti-diabetic activities [18]. In addition to isoflavone, soybean contains a variety of other biologically active compounds, including small peptides, soyasaponins, and phenolic acids [19]. Previously, we reported that the anti-oxidative stress and anti-inflammatory effects observed in adipose tissues of mice fed an HF diet containing *doenjang* were more potent than those in mice fed an HF diet containing steamed soybean [20]. Therefore, in the current study, we investigated whether *doenjang* and steamed soybean attenuate neuroinflammation and neurodegenerative characteristics in the cortex and hippocampus of mice fed an HF diet.

## 2. Materials and Methods 

### 2.1. Animals and Diets

Animal studies were approved by the Chungbuk National University Institutional Animal Care and Use Committee (CBNUA-636-13-01) and were described previously [20]. Briefly, male C57BL/6J mice (Nara Biotech Co., Seoul, Korea) at 4 weeks of age were acclimated for 1 week and were randomly allocated into four experimental diets (Unifaith Inc., Seoul, Korea) for 11 weeks. Four groups were fed a low-fat (LF: 11.7 kcal% fat) diet (LF group, *n* = 12), an HF (45.2 kcal% fat and 1% cholesterol) diet (HF group, *n* = 12), an HF diet containing 11.7% freeze-dried steamed soybean (SS group, *n* = 12), and an HF diet containing 14.4% freeze-dried *doenjang* (DJ group, *n* = 11). To adjust the soy protein intake to the level of a DJ diet, 11.7% of steamed soybean was added in an SS diet. Macronutrient content in DJ and SS diets was adjusted to those in an HF diet. The composition of diets [20] and the manufacturing process of *doenjang* were previously described [21]. Food, calorie intake, and feed efficiency are presented in Supplementary Table S1. Animals were housed under controlled temperature (21 ± 2 °C) and humidity (50 ± 20%) conditions with a 12-h dark-light cycle, and were given ad libitum access to food and water. After overnight fasting, mice were sacrificed using CO_2_ asphyxiation. The right cortex and hippocampus were fixed in phosphate-buffered formalin for histological analysis and the left cortex and hippocampus were snap frozen in liquid nitrogen and stored at −80 °C until the analysis.

### 2.2. Brain Tissue Histologic Examination

Formalin-fixed brain tissue was processed into 4-µm-thick paraffin sections and stained with cresyl violet for histological evaluation. The morphology was observed under an Olympus BX50 microscope with using a DP-72 digital camera (Olympus, Tokyo, Japan) and the image was captured using Image-Pro Plus ver. 4.5 program (Media Cybernetics Inc., Rockville, MD, USA). 

### 2.3. Brain Lipid Peroxidation and Protein Carbonylation Measurement

To measure brain thiobarbituric acid reactive substances (TBARS) and carbonylated proteins, the cortex and hippocampus were homogenized in 5% (*w*/*v*) of homogenizing buffer containing 154 mmol/L KCl, 50 mmol/L Tris-HCl, and 1 mmol/L ethylenediaminetetraacetic acid (pH 7.4), and the homogenates were centrifuged at 600× *g* for 10 min at 4 °C to obtain a supernatant. TBARS levels were measured according to the method of Ohkawa et al. [22]. The absorbance of the butanol layer was measured at 532 nm using 1,1,3,3-tetraethoxypropane as a standard. The brain lipid peroxide level was expressed as malondialdehyde equivalents per milligram of protein. Protein carbonylation was detected through 2,4-dinitrophenol hydrazine derivatization of the carbonyl groups, as previously described [23]. The absorbance of adducts was measured at 370 nm and the protein carbonyl content was expressed as nanomoles of carbonyl per milligram of protein.

### 2.4. Protein Extraction and Immunoblotting 

The cortex and hippocampus (≈50 mg) were homogenized in 500 μL of ice-cold protein lysis buffer using the Tissue lyser system (Qiagen, Hilden, Germany). After centrifugation for 30 min at 10,000× *g* at 4 °C, the protein content of the supernatant was determined with a protein assay kit (Bio-Rad, Hercules, CA, USA). Fifteen micrograms of protein were loaded into the lanes of a sodium dodecyl sulfate-polyacrylamide gel electrophoresis gel, separated, and transferred to an Immobilon-P membrane (Milipore, Burlington, MA, USA). After blocking with 5% nonfat milk or bovine serum albumin in a tris buffered saline with Tween 20, membranes were probed with specific antibodies as follows: anti-Aβ (Santa Cruz Biotechnology, Dallas, TX, USA), anti-catalase (Abcam, Cambridge, U.K.), anti-C/EBP homologous protein (CHOP; Cell Signaling Technology, Danvers, MA, USA), anti-cleaved caspase-3 (Cell Signaling Technology), anti-cyclic adenosine monophosphate (cAMP) response element-binding protein (CREB; Cell Signaling Technology), anti-p-CREB (Cell Signaling Technology), anti-p-glycogen synthase kinase 3β (GSK3β; Cell Signaling Technology), anti-c-Jun N-terminal kinase (JNK; Cell Signaling Technology), anti-p-JNK (Cell Signaling Technology), anti-proliferating cell nuclear antigen (PCNA; Santa Cruz Biotechnology), anti-protein phosphatase 2 (PP2A; Santa Cruz Biotechnology), anti-methylated PP2A (Millipore, Burlington, MA, USA), anti-spectrin alpha chain (Millipore), anti-total tau (Invitrogen, Carlsbad, CA, USA), anti-dephosphorylated tau (Millipore), anti-p-tau at S422 (Invitrogen), or anti-ubiquitin (Ub; Santa Cruz Biotechnology). The membrane was then incubated with an IgG-peroxidase-conjugated secondary antibody for chemiluminescent detection. For a loading control, the membrane was stripped and reprobed with an anti-β-actin monoclonal antibody (Sigma, St. Louis, MO, USA). The band intensities were quantified using Quantity One software (Bio-Rad). 

### 2.5. Total RNA Extraction and Quantitative PCR Analyses 

Total RNA of the cortex and hippocampus was isolated using the RNAiso Plus (Takara Bio Inc., Shiga, Japan) according to the manufacturer’s instructions. cDNA was synthesized using 2 µg of total RNA with Superscript II Reverse Transcriptase (Invitrogen). mRNA levels were analyzed using quantitative PCR (qPCR) with a StepOne™ Real-Time PCR System (Applied Biosystems, Foster City, CA, USA) using the SYBR^®^ Green PCR Master Mix (Applied Biosystems). Mouse β-actin was used as a reference gene and relative gene expression levels were analyzed using the 2^−ΔΔ*C*t^ method. The primer sequences are described in Supplementary Table S2.

### 2.6. Statistical Analysis

Statistical analyses were performed using SPSS (ver. 23.0, SPSS Inc., Chicago, IL, USA). For all experiments, one-way ANOVA followed by Duncan’s multiple range test were used to assess statistical significance. Data were expressed as means ± SEMs and differences were considered statistically significant at *p* < 0.05.

## 3. Results

### 3.1. Effects of Doenjang on Neuronal Death and Neurogenesis in the Cortex and Hippocampus of Mice Fed a High-Fat Diet 

**Figure 1:**
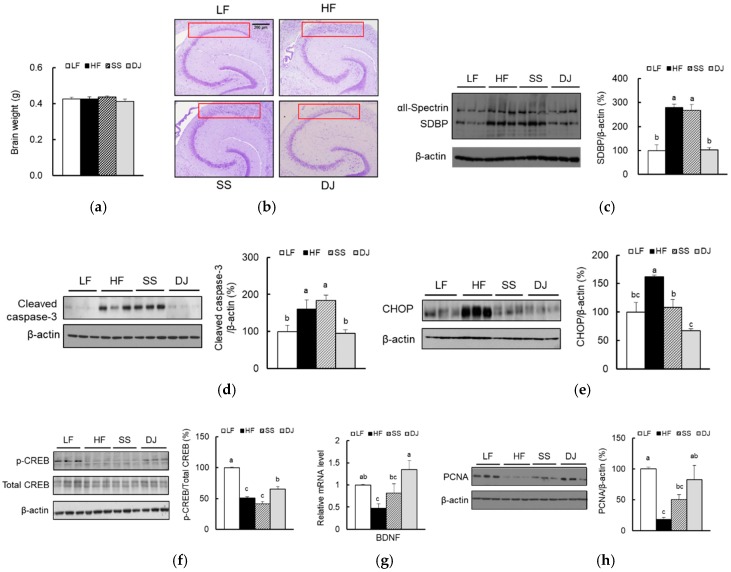
Effects of *doenjang* on neuronal death and neurogenesis in the cortex and hippocampus of mice fed a high-fat diet. (**a**) Brain weights (*n* = 11–12/group). (**b**) Representative hippocampal CA1 subfield sections stained with cresyl violet (*n* = 4/group). Relative protein levels of apoptotic markers: (**c**) SBDP, (**d**) cleaved caspase-3, and (**e**) CHOP were determined using immunoblotting (*n* = 3/group). (**f**) Relative protein levels of a neurogenesis marker, p-CREB, were determined using immunoblotting (*n* = 3/group). (**g**) Relative mRNA expression of a gene involved in synaptic plasticity, BDNF, were determined using qPCR (*n* = 4–5/group). (**h**) Relative protein levels of a proliferation marker, PCNA, were determined using immunoblotting (*n* = 3/group). Each bar represents the mean ± SEM. Data with a different alphabetical superscript were significantly different from one another (*p* < 0.05). LF, a low-fat diet; HF, a high-fat diet; SS, an HF diet containing freeze-dried steamed soybean; DJ, an HF diet containing freeze-dried *doenjang*; BDNF, brain-derived neurotrophic factor; CHOP, C/EBP homologous protein; CREB, cAMP-response element binding protein; SDBP, spectrin α breakdown products.

Previously, we have reported that an addition of *doenjang*, but not steamed soybean, significantly reduced body weight and adipose tissue weight of mice fed an HF diet for 11 weeks [20]. Significant difference was not observed in the brain weight of mice among the groups (Figure 1a). To determine whether HF feeding induced brain damage, we stained the hippocampus using cresyl violet and observed neuronal loss in the hippocampal CA1 region in mice fed an HF diet compared to mice fed an LF diet (Figure 1b). These alterations were alleviated in the DJ group, but not in the SS group. To confirm whether feeding mice an HF diet induced neuronal death, we measured spectrin α breakdown products (SBDP). The calpain or caspase-3 modulate cleavage of α-spectrin, resulting in increases in breakdown products, SBDP [24]. Consumption of *doenjang* significantly reduced SBDP levels (Figure 1c). Additionally, we observed that protein levels of cleaved caspase-3 and C/EBP homologous protein (CHOP), an ER stress-mediated apoptosis marker, were significantly increased in the HF group (Figure 1d,e). Addition of *doenjang* significantly reduced both cleaved caspase-3 and CHOP levels to a significantly greater degree compared to steamed soybean. 

Because brain-derived neurotrophic factor (BDNF) is shown to regulate neurogenesis [25], we measured whether the addition of *doenjang* regulates the CREB-BDNF pathway. Indeed, HF feeding significantly inactivated CREB and downregulated BDNF mRNA levels, which were significantly reversed by *doenjang* (Figure 1f,g). Furthermore, *Doenjang* increased neurogenesis based on proliferation cell nuclear antigen (PCNA) protein levels (Figure 1h). These results suggest that *doenjang* significantly attenuated neuronal apoptosis and improved adult neurogenesis.

### 3.2. Effects of Doenjang on Oxidative Stress and Neuroinflammation in the Cortex and Hippocampus of Mice Fed a High-Fat Diet

**Figure 2:**
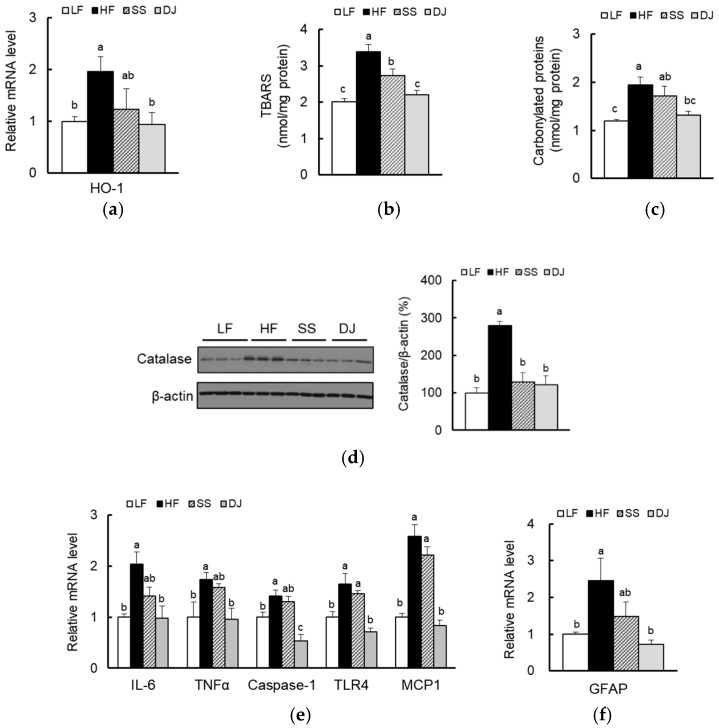
Effects of *doenjang* on oxidative stress and neuroinflammation in the cortex and hippocampus of mice fed a high-fat diet. (**a**) Relative mRNA expression of genes involved in oxidative stress, HO-1, was determined using qPCR (*n* = 4–5/group). Levels of (**b**) TBARS and (**c**) carbonylated proteins were determined using enzymatic assays (*n* = 5/group). (**d**) Relative protein levels of catalase were determined using immunoblotting (*n* = 3/group). Relative mRNA expression of genes involved in (**e**) pro-inflammation and in (**f**) astrocyte activation were determined using qPCR (*n* = 4–5/group). Each bar represents the mean ± SEM. Data with a different alphabetical superscript were significantly different from one another (*p* < 0.05). LF, a low-fat diet; HF, a high-fat diet; SS, an HF diet containing freeze-dried steamed soybean; DJ, an HF diet containing freeze-dried *doenjang*; GFAP, glial fibrillary acidic protein

Oxidative stress could lead to increased inflammatory cytokine levels, memory loss, and neuronal apoptosis [26,27]. Because *doenjang* is known to reduce oxidative stress by regulation of antioxidant enzymes [20], we measured several oxidative stress markers. Expression of heme oxygenase 1 (HO-1) was significantly induced in mice fed an HF diet, which were reduced in mice fed a DJ diet (Figure 2a). Furthermore, significant increases in TBARS and carbonylated protein contents were observed in mice fed an HF diet (Figure 2b,c). Consumption of *doenjang* significantly reduced both TBARS and carbonylated protein contents. Lesser effects were observed in mice fed an SS diet. Significantly lower catalase protein levels were observed in both SS and DJ groups compared to an HF group (Figure 2d).

In response to peripheral inflammatory signaling, several cell types in the brain undergo phenotypic changes, resulting in an activation of the nuclear factor kappa-light-chain-enhancer of activated B cells (NF-κB)-mediated signaling pathway in the HF diet-fed animals. Furthermore, obesity-mediated neuroinflammation is identified by upregulated gene expression of cytokines and chemokines [28]. Consistent with previous studies, the mRNA levels of genes involved in neuroinflammation, such as interleukin 6 (IL-6), tumor necrosis factor alpha (TNFα), caspase 1, Toll-like receptor 4 (TLR4), and monocyte chemoattractant protein 1 (MCP1), were significantly upregulated in mice fed an HF diet. Significant decreases of proinflammatory gene expressions were observed in the cortex and hippocampus of mice fed a DJ diet, but not in mice fed an SS diet (Figure 2e). We also determined whether activated astrocytes were involved in the upregulation of inflammatory cytokines and oxidative stress. In the DJ group, mRNA levels of glial fibrillary acidic protein (GFAP), one of the major intermediate filament proteins of activated astrocytes, were significantly lower compared to the HF group (Figure 2f).

### 3.3. Effects of Doenjang on β-amyloid Deposition in the Cortex and Hippocampus of Mice Fed a High-Fat Diet

**Figure 3:**
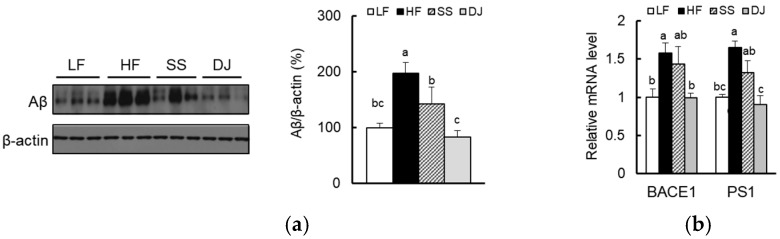

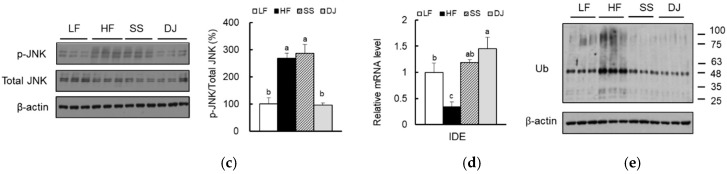
Effects of *doenjang* on Aβ deposition in the cortex and hippocampus of mice fed a high-fat diet. (**a**) Relative protein levels of Aβ were determined using immunoblotting (*n* = 3/group). (**b**) Relative mRNA expression of genes involved in Aβ production, BACE1 and PS1, were determined using qPCR (*n* = 4–5/group). (**c**) Relative protein levels of APP kinase, JNK, were determined using immunoblotting (*n* = 3/group). (**d**) Relative mRNA expression of a gene involved in Aβ degradation, IDE, were determined using qPCR (*n* = 4–5/group). (**e**) Relative ubiquitinated protein levels were determined using immunoblotting (*n* = 3/group). Each bar represents the mean ± SEM. Data with a different alphabetical superscript were significantly different from one another (*p* < 0.05). LF, a low-fat diet; HF, a high-fat diet; SS, an HF diet containing freeze-dried steamed soybean; DJ, an HF diet containing freeze-dried *doenjang*; IDE, insulin degrading enzyme.

Aβ deposition was significantly reduced by both *doenjang* and steamed soybean consumption (Figure 3a). Aβ is a peptide of 40 or 42 amino acids derived predominantly from amyloid precursor protein (APP) upon sequential cleavage by β-secretase 1 (BACE1) and γ-secretase [29]. An HF diet significantly increased expressions of BACE1 and presenilin 1 (PS1), a crucial component of γ-secretase. Addition of *doenjang*, but not steamed soybean, significantly downregulated mRNA levels of BACE1 and PS1 in mice fed an HF diet (Figure 3b). JNK is known to regulate phosphorylation and amyloidogenic cleavage of APP [30]. We observed that JNK was significantly inactivated by the addition of *doenjang* in HF diet-fed mice (Figure 3c). Moreover, expressions of the insulin degrading enzyme (IDE), which is involved in the degradation of Aβ as well as insulin, were significantly upregulated by the addition of steamed soybean and *doenjang* (Figure 3d). These results indicate that *doenjang* decreased Aβ accumulation through suppressing the amyloidogenic pathway and increasing IDE-mediated clearance. To determine whether the accumulated Aβ was caused by dysfunction of the ubiquitin/proteasome system, we measured ubiquitinated protein levels using immunoblotting (Figure 3e). Addition of *doenjang* and steamed soybean markedly reduced ubiquitinated protein levels.

### 3.4. Effects of Doenjang on Tau Hyperphosphorylation in Cortex and Hippocampus of Mice Fed a High-Fat Diet

**Figure 4:**
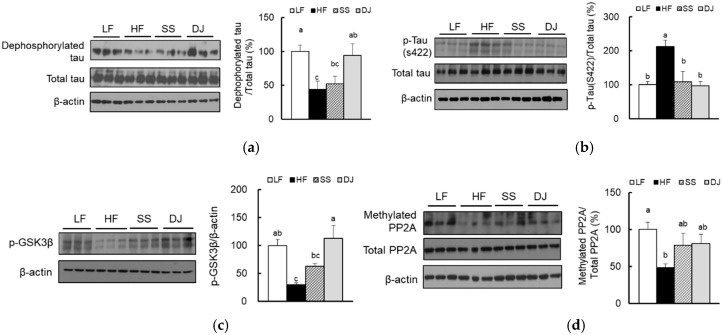
Effects of *doenjang* on tau hyperphosphorylation in the cortex and hippocampus of mice fed on a high-fat diet. Relative protein levels of (**a**) dephosphorylated tau, (**b**) phosphorylated tau (ser422), (**c**) phosphorylated tau kinase, GSK3β, and (**d**) methylated tau phosphatase, PP2A, were determined using immunoblotting (*n* = 3/group). Each bar represents the mean ± SEM. Data with a different alphabetical superscript were significantly different from one another (*p* < 0.05). LF, a low-fat diet; HF, a high-fat diet; SS, an HF diet containing freeze-dried steamed soybean; DJ, an HF diet containing freeze-dried *doenjang*.

We observed that the dephosphorylated form of tau was reduced by HF feeding, indicating an increased tau hyperphosphorylation in the cortex and hippocampus (Figure 4a). Protein levels of hyperphosphorylated tau in HF diet-fed mice were significantly reduced by the addition of *doenjang*. Phosphorylation of tau at serine 422, which is associated with intracellular and extracellular neurofibrillary structures, was significantly lower in the SS and DJ groups compared to the HF group (Figure 4b). Accordingly, *doenjang* consumption significantly inactivated glycogen synthase kinase (GSK)-3β, one of major tau kinases, by increasing p-GSK3β levels (Figure 4c). In contrast, a decreased catalytic activity of tau phosphatase, determined using the methylation status of protein phosphatase 2A (PP2A) at leucine 309, was not significantly changed by the consumption of *doenjang* or steamed soybean (Figure 4d).

## 4. Discussion

Previously, we reported that *doenjang* has a higher potential for reducing obesity and ameliorating inflammation and oxidative stress in the adipose tissue of obese mice than steamed soybean [20]. Therefore, the protective effect of *doenjang* against neuronal degeneration in mice fed an HF diet was investigated in the present study. It has been well documented that oxidative stress is crucial for the inflammatory cytokine release and cognitive dysfunction [31]. Based on the above considerations, we determined several molecular markers of brain in Alzheimer’s disease and observed that the consumption of an HF diet induced oxidative stress, neuronal inflammation, Aβ accumulation, tau hyperphosphorylation, and neuronal cell death, which were significantly alleviated by the consumption of *doenjang*. Although we did not determine in vivo cognition and memory function, the neuroprotective effects of steamed soybean in most parameters determined in the present study was less potent compared to those of *doenjang*.

Much evidence from in vitro and in vivo models indicates that isoflavone exerts the neuroprotective and neurotrophic effects. However, this is the first study that did compare the effect of soybean and its fermented form in a diet-induced neurodegenerative disease model. In line with our observations, consumption of *chungkookjang*, a traditional Korean short-term fermented soybean food, significantly decreased Aβ deposition compared to non-fermented cooked soybeans in rats with partial pancreatectomy and intracerebroventricular infusion of Aβ 25−35 [32]. In a similar fashion, *chungkookjang* was more effective than cooked soybean in reducing hippocampal cell death in gerbils with transient artery occlusion [33]. Furthermore, one-time oral administration of total isoflavone (40 mg/kg) from *tempeh*, a traditional Indonesian fermented soybean food, was able to improve cholinergic activities and reduce some inflammation markers better than total isoflavone from soybean in scopolamine induced amnesia rats [34]. The combination of genistein plus daidzein plus equol showed a higher binding selectivity for estrogen receptor β and a greater efficacy compared to a single formulation [35], indicating that fermented soybean food could be a part of an overall healthy diet designed to lower the risk of neurodegenerative diseases. The content of isoflavone aglycone was about 10 times higher in a DJ diet compared to an SS diet (152.64 vs. 15.21 mg/kg diet) [20,21]. When male rats were fed a diet containing 100 and 5 mg genistein/kg, serum total genistein concentrations were shown to reach to 0.59 and 0.06 μmol/L, respectively [36]. Several previous studies have reported gender-related differences in activities of conjugating enzymes and β-glucosidase, and urinary recovery and excretion half-life of isoflavone [37]. Furthermore, effects of dietary isoflavone on oxidative DNA damage in the blood were gender-dependent in a human intervention study [38]. Therefore, further research is needed to investigate the gender-related differences of fermented soybean foods in cognitive function.

As expected, consumption of *doenjang* prevented neuroinflammation and neurodegeneration through inhibition of the oxidative modifications and upregulation of endogenous antioxidant signaling pathways. Furthermore, expressions of estrogen-receptor-responsive genes, including BDNF and IDE, were significantly upregulated in mice fed a DJ diet. Especially, we observed a significantly higher level of neurogenesis in mice fed a DJ diet compared to mice fed an SS diet. In agreement with previous studies reporting that CREB phosphorylation and BDNF expression are regulated by estrogen-receptor mediated manners [39,40], our data showed that increased levels of isoflavone aglycone may be involved in improving synaptic plasticity and defending neurodegenerative diseases. Similarly, *chungkookjang* stimulated nerve growth factor secretion and nerve growth factor receptor signaling pathway in Tg2576 mice [41] and in trimethyltin-induced cognitive defective mice [42]. In addition to isoflavone, soyasaponins are shown to prevent scopolamine-induced memory impairment in mice by preserving BDNF expression and CREB phosphorylation [43]. 

## 5. Conclusions

Collectively, our study demonstrated that *doenjang* alleviated the inflammatory response and oxidative stress in cortex and hippocampus of mice fed an HF diet. *Doenjang* also improved synaptic plasticity and decreased synaptic loss, which were accompanied by altered Aβ accumulation and tau hyperphosphorylation. Moreover, the overall neuroprotective effects of *doenjang* were significantly higher compared to steamed soybean in the cortex and hippocampus of mice. Further studies are needed to address whether fermented soybean foods containing various bioactive compounds could be beneficial toward alleviating obesity-mediated neuronal damage in the adult brain.

## Supplementary Materials

The following are available online at https://www.mdpi.com/2072-6643/11/8/1702/s1, Table S1: Effects of *Doenjang* on food intake and feed efficiency ratio of mice, Table S2: List of primer sequences for quantitative RT-PCR (qRT-PCR).
