# Supplementary material for: Doenjang, A Korean Traditional Fermented Soybean Paste, Ameliorates Neuroinflammation and Neurodegeneration in Mice Fed a High-Fat Diet
Source: Nutrients. 2019 Jul 24;11(8):1702. doi: 10.3390/nu11081702 (PMC6723205; doi:10.3390/nu11081702)
Supplement: Supplementary file 1 [file nutrients-11-01702-s001.pdf]

Supplementary Table S1. Effects of *Doenjang* on food intake and feed efficiency ratio of mice

	LF	HF	SS	DJ
Food intake (g/day)	3.93± 0.09 [a]	2.60 ± 0.08 [b]	2.69 ± 0.20 [b]	2.47 ± 0.08 [b]
Calorie intake (kcal/day)	15.12 ± 0.35 [a]	12.35 ± 0.37 [b]	12.60 ± 0.98[b]	11.21 ± 0.33[b]
Feed efficiency ratio (%)	3.72 ± 0.32 [c]	8.08 ± 0.53 [ab]	9.77 ± 0.85[a]	6.64 ± 0.58[b]

LF, a low-fat diet (n = 12/group); HF, a high-fat diet (n = 12/group); SS, an HF diet containing steamed soybean (n = 12/group); DJ, an HF diet containing *Doenjang* (n = 11/group). Values are mean ± SEM. Data in the same row with different alphabetical superscript are significantly different from one another ($P < 0.05$).

Supplementary Table S2. List of primer sequences for quantitative RT-PCR (qRT-PCR)

Gene	Forward (5′- 3′)	Reverse (5′- 3′)
BACE1	GCATGATCATTGGTGGTATC	CCATCTTGAGATCTTGACCA
BDNF	GGCTGACACTTTTGAGCACGTC	CTCCAAAGGCACTTGACTGCTG
Caspase-1	GAATACAACCACTCGTACAC GTCTTG	AGATCCTCCAGCAGCAACTTCA
GFAP	AACCGCATCACCATTCCT	CGCATCTCCACAGTCTTTACC
HO-1	CCTCACTGGCAGGAAATCATC	CCTCGTGGAGACGCTTTACATA
IDE	CTGTGCCCCTTGTTTGATGC	GTTCCCCGTAGCCTTTTCCA
IL-6	TTCCATCCAGTTGCCTTCTT	CAGAATTGCCATTGCACAAC
MCP-1	TGATCCCAATGAGTAGGCTGG	ATGTCTGGACCCATTCCTTCTTG
PS1	TGCGGCCATCATGATCAGTGTC	ATAAGCCAGGCGTGGATGAC
TLR-4	AGGAAGTTTCTCTGGACTAACAAG TTTAGA	AAATTGTGAGCCACATTGAGTTTC
TNFα	GGCTACAGGCTTGRCACTCGA	CACGCTCTTCTGTCTACTGAA
β-Actin	GCTGAGAGGGAAATCGT	CGTCAGGCAGCTCATAG

BACE1, β-secretase 1; BDNF, brain-derived neurotrophic factor; GFAP, glial fibrillary acidic protein; HO-1, heme oxygenase 1; IDE, insulin-degrading enzyme; IL-6, interleukin 6; MCP-1, monocyte chemoattractant protein-1; PS1, presenilin 1; TLR-4, Toll-like receptor 4; TNFα, tumor necrosis factor α
